# Supplemental breast cancer-screening ultrasonography in women with dense breasts: a systematic review and meta-analysis

**DOI:** 10.1038/s41416-020-0928-1

**Published:** 2020-06-12

**Authors:** Wei-Hsin Yuan, Hui-Chen Hsu, Ying-Yuan Chen, Chia-Hung Wu

**Affiliations:** 1 Division of Radiology, Taipei Municipal Gan-Dau Hospital (Managed by Taipei Veterans General Hospital), Taipei, Taiwan, ROC; 2 School of Medicine, National Yang-Ming University, Taipei, Taiwan, ROC; 3 Department of Radiology, Taipei Veterans General Hospital, Taipei, Taiwan, ROC; 4 Department of Medical Imaging, Taiwan Adventist Hospital, Taipei, Taiwan, ROC; 5 Division of Radiology, National Yang-Ming University Hospital, Yilan City, Taiwan, ROC

**Keywords:** Breast cancer, Cancer

## Abstract

**Background:**

Mammography is not effective in detecting breast cancer in dense breasts.

**Methods:**

A search in Medline, Cochrane, EMBASE and Google Scholar databases was conducted from January 1, 1980 to April 10, 2019 to identify women with dense breasts screened by mammography (M) and/or ultrasound (US). Meta-analysis was performed using the random-effect model.

**Results:**

A total of 21 studies were included. The pooled sensitivity values of M alone and M + US in patients were 74% and 96%, while specificity of the two methods were 93% and 87%, respectively. Screening sensitivity was significantly higher in M + US than M alone (risk ratio: M alone vs. M + US = 0.699, *P* < 0.001), but the slight difference in specificity was statistically significant (risk ratio = 1.060, *P* = 0.001). Pooled diagnostic performance of follow-up US after initial negative mammography demonstrated a high pooled sensitivity (96%) and specificity (88%). The findings were supported by subgroup analysis stratified by study country, US method and timing of US.

**Conclusions:**

Breast cancer screening by supplemental US among women with dense breasts shows added detection sensitivity compared with M alone. However, US slightly decreased the diagnostic specificity for breast cancer. The cost-effectiveness of supplemental US in detecting malignancy in dense breasts should be considered additionally.

## Background

Mammography has been established as the primary method of screening for breast cancer, and since its introduction, the diagnosis of early-stage disease was significantly enhanced.^1,2^ The overall sensitivity of mammography for the detection of non-palpable cancers is approximately 85%,^1,2^ but the density of breast tissue can markedly reduce the mammography detection rate of early-stage disease.^3–5^ Breast density can be classified according to the Breast Imaging-Reporting and Data System (BI-RADS) American College of Radiology (ACR) categories and/or quantification: BI-RADS A: almost entirely fat (low density of mammary gland parenchyma), BI-RADS B: scattered fibroglandular densities (average density of gland parenchyma), BI-RADS C: heterogeneously dense (high density of gland parenchyma) and BI-RADS D: extremely dense (very high density of gland parenchyma).^6,7^ In women with >75% breast parenchymal dense tissue, the sensitivity of mammography for detecting early-stage cancer can be as low as 48%.^4,5^ Dense breast tissue is an independent marker associated with increased breast cancer risk, especially in women who are at higher risk due to other factors such as family history.^8^ Women with dense breast tissue who develop carcinoma in one breast are also at higher risk of developing cancer in the contralateral breast.^9^ It is estimated that approximately two-thirds of premenopausal women and one-third of elderly women aged 75–79 years have a breast density of 50% or higher.^4^ Furthermore, ethnic differences also exist as dense breasts are more prevalent in Asian than in Caucasian women.^10,11^ Although breast cancer incidence rates in the Asian population were found to be lower than those in the Western population according to a large-scale epidemiology study, the incidence in Asia is quickly increasing and surpassing than in the Western countries.^12^ This highlights an urgent need for more efficient breast cancer prevention and management strategies in the Asian population.

In light of the limitations of mammography in women with dense breasts, a study has suggested that ultrasound (US) is more sensitive than mammography, and can identify mammography- occult breast cancers in dense breasts, especially of younger women aged 30–39.^13^ Other studies have indicated that adjunctive US and mammography in women with dense breasts resulted in a significant increase in the cancer detection rate as compared with mammography alone.^3,14^ Some authors have therefore suggested that mammography with supplemental US screening can be beneficial for women with dense breasts, specific female groups prone to have dense breast tissue as previously described and women in resource-poor healthcare systems.^15^ In the prospective J-START study, improved sensitivity in breast cancer detection was found in mammography with ultrasound compared with without (91.1% vs. 77.0%) in asymptomatic Japanese women aged 40–49 years unlimited to breast density, albeit with a concurrent lower specificity.^16^ In particular, breast cancers detected by US are likely to be different characteristically than those detected by mammography, in which breast cancers detected by US are more likely to be smaller-sized, invasive and of the luminal A subtype compared with those detected by mammogram.^17^ Nevertheless, the use of adjunctive US may increase the number of false-positive findings and unnecessary biopsy recommendations,^18,19^ and the added diagnostic benefit of the screening strategy should be reconsidered as a whole.

Therefore, the added clinical benefit of US to mammography has been the interest of many systematic reviews, which provides only qualitative evaluation of published clinical evidence. In contrast, quantitative analysis that pooled and compared the diagnostic yield of conjunctive or sequential mammography and US screening strategies is limited. Moreover, it was suggested by a systematic review that the overall available evidence regarding the detection rate of breast cancer by screening with mammography and adjunct US may be low based on the Grades of Recommendation Assessment Development and Evaluation (GRADE) system.^18^

Thus, the objective of this study was to perform a systematic review and meta-analysis examining the diagnostic performance of mammography alone plus US for breast cancer in women with dense breasts, as well as that of follow-up US in women with dense breasts and negative mammography results.

## Methods

### Literature search and study selection

This meta-analysis was performed in accordance with the PRISMA guidelines,^20^ and the PICO model for clinical questions (P: Patient, Population or Problem [Female patients with heterogeneously or extremely dense breasts], I: Intervention, Prognostic Factor or Exposure [Screening mammography in combination with ultrasonography or ultrasonography as adjuvant for mammography-negative women], C: Comparison or Intervention [Only screening mammography or no comparative group] and O: Outcome Measures [sensitivity, specificity, positive predictive value [PPV], negative predictive value [NPV] and accuracy for detecting early-stage breast cancer). The review protocol had not been registered or published previously.

Medline, Cochrane, EMBASE and Google Scholar databases were searched for studies published between January 1, 1980 and April 10, 2019 using the keywords as follows: breast, dense, density, breast cancer, breast density, mammography, ultrasonography, ultrasound, specificity, sensitivity, screening and comparison. The search strategies included “*mammography and ultrasound and breast and (dense OR density) with search filters of abstract availability, publications English, and clinical trials*”, and “*(ultrasound) AND (mammography) AND (breast cancer) AND (screening) AND (sensitivity) AND (specificity) AND (comparison)*”. Literature inclusion criteria for meta-analysis were (1) randomised controlled trials (RCTs), 2-arm prospective studies, retrospective studies and cohort studies; (2) participants were women with dense breasts with BI-RADS categories ≥2; (3) study design included either mammography with adjunctive ultrasonography or additional ultrasonography following a negative mammography; (4) quantitative outcome data for outcomes of interest (i.e. PPV, NPV, sensitivity and specificity); (5) full-text studies published in English. Letters, comments, editorials, case reports, proceedings and personal communication were excluded. Studies of patients without dense breasts, and those that did not provide direct comparisons of the outcomes of interest, were further excluded. Studies designed for the detection of microcalcifications were excluded due to the technical nature of ultrasound, which is limited to detect breast microcalcifications.^21,22^ The reference lists of articles included for qualitative review were searched for studies that fit the above criteria. Literature searches were performed by two independent reviewers who were breast cancer specialists, and a third reviewer, also a breast cancer specialist, was consulted for resolutions of any disagreements.

### Quality assessment

The quality of the included studies was assessed using QUADAS-2, a revised tool for the quality assessment of diagnostic accuracy studies.^23^ Briefly, QUADAS-2 comprises four domains: patient selection, index test, reference standard and flow and timing. Each domain was assessed for risk of bias, and the first three domains were subsequently assessed regarding topic relevance.

### Data extraction and statistical analysis

Studies’ characteristics, including the number of total enrolled patients and number of patients with confirmed cancer, mean ± standard deviation (SD), mean or median with range (minimum–maximum) for age, detection rate per 1000 patients screened in cancer detection or added cancer detection benefit, were extracted. The dispersion of density categories, definition of dense breast, recall rate, biopsy rate per 1000 patients screened, reference standard and PPV were also extracted and summarised in preformed data forms accordingly. PPV1 was defined as the malignancy rate among cases with positive results; PPV2 was defined as the malignancy rate among positive cases with biopsy recommendations; PPV3 was defined as the malignancy rate of positive cases with a performed biopsy. The diagnostic outcomes, including sensitivity and specificity for the detection of early-stage breast cancer, were extracted according to full-text reviewing, and summarised as % (TP/TP + FN) and % (TN/FP + TN), respectively, where TP, FP, TN and FN indicated the number of patients with true positivity, false positivity, true negativity and false negativity predicted. Specificity, sensitivity or the difference between these outcomes where available were further evaluated by meta-analysis.

Through Meta-DiSc analysis, sensitivity and specificity of cancer detection from either test arm were then calculated and summarised as a forest plot presenting values of each study with the corresponding 95% confidence interval (CI, lower and upper limit), and then a pooled effect among those studies with completed measurements was calculated. Furthermore, a summary receiver-operating characteristic (SROC) curve was graphed along with the area under SROC curve (AUC) with standard error (SE).

For comparing the differences in diagnostic performance between mammography alone (M alone) and mammography with conjunctive ultrasound (M + US) in dense breast patients, an effect size defined as risk ratio (RR) was adopted and presented with 95% CI for each study, and a combined effect was subsequently calculated using the Comprehensive Meta-Analysis software, version 2.0 (Biostat, Englewood, NJ). An RR > 1 indicated that M alone might provide a higher diagnostic value than M + US, while an RR < 1 indicated that M + US provided a higher diagnostic value than M alone. An RR = 1 indicated that the results were similar between M alone and M + US.

The heterogeneity test was evaluated according to a χ^2^-based statistic and *I*^2^ statistic with a p value. For the Q statistic (or otherwise indicated as chi-square), *P* values <0.10 were considered statistically significant for heterogeneity. For the *I*^2^ statistic, heterogeneity was assessed as follows: no heterogeneity (*I*^2^ = 0–25%), moderate heterogeneity (*I*^2^ = 25–50%), large heterogeneity (*I*^2^ = 50–75%) and extreme heterogeneity (*I*^2^ = 75–100%).^24^ A random-effect model was used in the current meta-analysis, assuming substantial heterogeneity present among the studies.^25^

Subgroup analyses were performed with regard to study country, US method and available data obtained during first-round US. Sensitivity analysis was conducted using a leave-one-out approach. Publication bias analysis by funnel plot was not performed in the current meta-analysis due to the limited number of studies included (<10 studies).^26^ In all analyses, a two-sided *P* value <0.05 was considered statistically significant. The statistical analyses were performed using Meta-DiSc analysis software, version 1.4 and Comprehensive Meta-Analysis software, version 2.0 (Biostat, Englewood, NJ).

## Results

### Literature search

**Fig. 1:**
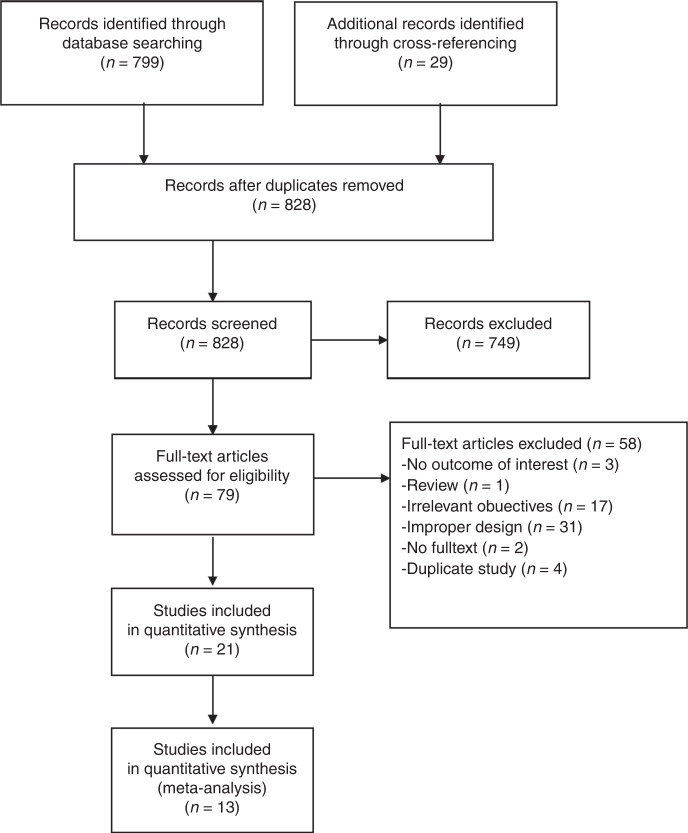
Flow diagram of study selection for systematic review and meta-analysis. Twenty-one studies with quantitative synthesis were included for systematic review and 13 studies with complete diagnostic results were included for conducting meta-analysis.

A flow diagram of study selection is shown in Fig. 1. After initially identifying 828 articles, 749 articles were excluded based on the exclusion criteria. The full text of 79 articles was then reviewed, and 58 articles were excluded; the reasons for exclusion are shown in Fig. 1. Three sets of studies (Corsetti et al.,^27,28^ Berg et al.^29,30^ and Weigert et al.^31–33^) were series reports of three individual patient cohorts. The earlier papers were excluded due to data duplication or lack of data on sensitivity and specificity. Thus, 21 studies were finally included in a systematic review.^3,28,30,33–50^

### Study characteristics

Study characteristics are summarised in Table 1; recall rate, biopsy rate, PPV1 and PPV3 are summarised in Table 2. The risk factors considered in each of the studies were summarised in Supplementary Table S1. Eight studies included women with dense breasts who received M alone or M + US screening for breast cancer,^3,30,34,36,40,42,47,49^ and five of these performed US using the automated breast US (ABUS).^3,34,40,42,49^ US was done in a whole-breast screening fashion in all included studies. In these studies, 443 out of 69,096 participants were diagnosed with malignancies confirmed by biopsy. Most of the studies that compared M alone with M + US were performed in countries highly populated by Caucasians (United States and Sweden).^3,30,34,40,42,49^ Five of the eight studies comparing M alone and M + US in patients with dense breasts provided data for the presence of common breast cancer risk factor, which included BRCA1/2 mutations, family history, personal breast cancer history, use of hormone therapy, etc.^3,30,34,42,49^ (Supplementary Table S1).

**Table 1:** Characteristic of studies included in the meta-analysis.

First author	Study design	Country	Ethnicity (%)	US method	Comparison	Number of patients	Age (years)^a^	Cancer detection rate (per 1000 screened)	Added cancer detection rate (per 1000 screened)	Number of patients with confirmed cancer
*Patients with dense breasts*										
Wilczek et al.^49^	Retro	Sweden	Not specified	ABUS (Rg)	M alone	1668	49.5 ± 7.9	4.2	2.4	11
				(Rg)	M + US	1668		6.6		
Brem et al.^34^	Pro	USA	White 11915 (77.8) Hispanics, Latinas or Spanish 810 (5.3) American Indians or Alaskan natives 33 (0.2) Asians 657 (4.3) Blacks, African Americans or Haitians only 1645 (10.7) Native Hawaiians or Pacific Islanders 27 (0.2) Mixed race or ethnicity 108 (0.7) Unknown or other race or ethnicity 123 (0.8)	ABUS	M alone	15,318	53.3 ± 10.0	5.4	1.9	82
				(T)	M + US			7.3		112
Giger et al.^40^^b^	Retro	USA	Not specified	ABUS	M alone	185	N/A	N/A	N/A	52
				(R)	M + US	185	N/A	N/A		
Giuliano^42^	Pro	USA	Not specified	ABUS	M alone	4076	Median 54^c^	4.6	7.7	19
				(T)	M + US	3418	Median 57^c^	12.3		42
Korpraphong et al.^47^	Retro	Thailand	Not specified	HHUS (R)	M alone	14,483	Mean 49.4	6.5	1.4	115
					M + US			7.9		
Chae et al.^36^	Retro	Korea	Asian	HHUS (R)	M alone	12,505	Mean = 52 (range 22–91)	0.48	2.391	11
					M + US	8359		2.871		24
Berg et al.^30^	RCT	USA	White 2467 (92.8) Hispanic or Latino 265 (10.0) Black or African American 91 (3.4) Native Hawaiian or other Pacific Islander 4 (0.2) Asian 90 (3.4) American Indian or Alaskan Native 4 (0.2) Unknown 11 (0.4)	HHUS (P)	M alone	2659	55.2 ± 10.1	7.5	5.3	N/A
					M + US			12.8		
Kelly et al.^3^	Pro	USA	White 3843 (87) Hispanics 248 (6) Asians 128 (3) Blacks 61 (1)	ABUS (T)	M alone	6425	Median = 53 (range, 24–89)	3.6	3.6	57
					M + US	6425		7.2		
*Patients with negative mammogram and dense breasts*										
Destounis et al.^39^	Retro	USA	Not specified	HHUS	M (–) → US	4898	Mean 55.8	3.3	N/A	18
Klevos et al.^46^	Retro	USA	Not specified	HHUS (T followed by a radiologist)	M (–) → US	394	NA	N/A	N/A	0
Kim et al.^45^	Pro	Korea	Asian	HHUS (R)	M (–) → US	Total: 3,171 Initial US: 998 Non-initial US: 2173	51.2 ± 7.7	Total: 2.8 Initial US: 4 Non-initial US: 2.3	N/A	Total: 9 Initial US: 4 Non-initial US: 5
Weigert^33^	Retro	USA	Not specified	HHUS (T, in some substances, radiologist-rescanned)	M (–) → US	Year 1: 2706 Year 2: 3351 Year 3: 4128 Year 4: 3331	Range 45–77	Year 1: 4.1 Year 2: 2.7 Year 3: 2.7 Year 4: 3.0	N/A	Year 1: 11 Year 2: 9 Year 3: 11 Year 4: 10
Chang et al.^37^	Retro	Korea	Asian	HHUS (R)	M (–) → US	990	Median = 47 (range, 27–79)	5.1	N/A	5
Girardi et al.^41^	Retro	Italy	Not specified	HHUS (R)	M (–) → US	9960	NA	2.21	N/A	22
Hooley et al.^43^	Retro	USA	Not specified	HHUS (T)	M (–) → US	935	52 (range, 29–86)	3.2	N/A	3
Leong et al.^48^	Pro	Singapore	Chinese (94) Indian (4) Malay (1) Eurasian (1)	HHUS (S, radiologist verified)	M (–) → US	141	Mean = 45.1 (range, 30–64)	US: 14	N/A	2
Corsetti et al.^28^	Retro	Italy	Not specified	HHUS	M (–) → US	3356	N/A	US: 4.4	N/A	N/A
Youk et al.^50^	Retro	Korea	Asian	HHUS (R)	M (–) → US	1507	47.5 ± 7.8	29	N/A	43
Crystal et al.^38^	Retro	Israel	N/A	HHUS (R)	M (–) → US	1517	52.1 ± 8.1	4.6	N/A	7
Kaplan^44^	Pro	USA	Not specified	HHUS (T or R)	M (–) → US	1862	Range 35–87	3	N/A	6
Buchberger et al.^35^	Retro	Austria	Not specified	HHUS (R)	M (–) → US	8103	Mean 47.6	4.1	N/A	40

*ABUS* automated breast US, *HHUS* handheld US, *M* mammogram, *N/A* not available, *Pro* prospective study, *Retro* retrospective study, *RCT* randomised controlled trial, *US* ultrasound, *USA* United States of America, *R* radiologist-performed, *Rg* radiographer-performed, *P* physician-performed, *S* sonographer-performed, *T* technologist-performed.

^a^Age was summarised as mean ± standard deviation, or mean or median with range (minimum, maximum).

^b^Enriched-reader study.

^c^Patients with cancer.

**Table 2:** Summary of recall rate, biopsy rate and biopsy-referenced PPV.

First author	Comparison	Recall rate	Biopsy rate (per 1000)	Reference standard	PPV3	PPV1
*Patients with dense breasts*						
Wilczek et al.^49^	M alone	13.8/1000	6.6	Biopsy result	63.6% (7/11)	30.4% (7/23)
	M plus US	22.8/1000	13.8		47.8% (11/23)	28.9% (11/38)
Brem et al.^34^	M alone	2301/15,318	38.3	Biopsy result	14% (82/586)	3.6% (82/2301)
	M plus US	4364/15,318	74.3		9.8% (112/1138)	2.6% (112/4364)
Giger et al.^40^	M alone		N/A	Biopsy result	N/A	50.85% (30/59)
	M plus US		N/A		N/A	55.07% (38/69)
Giuliano^42^	M alone		4.6	Biopsy result	N/A	20.43% (19/93)
	M plus US		12.3		N/A	80.77% (42/52)
Korpraphong et al.^47^	M alone		6.5	Biopsy result	N/A	35% (105/300)
	M plus US		7.9		N/A	20.2% (115/569)
Chae et al.^36^	M alone	4.20%	1	Biopsy result and 2-year follow-up	50% (6/12)^a^	1.14% (6/526)
	M plus US	5.50%	26		11.1% (24/216)^a^	5.30% (24/452)
Berg et al.^30^	M alone	11.5% (306/2659)	24	Biopsy result and 12-month follow-up	29.2% (19/65)	6.5% (20/306)
	M plus US	26.6% (707/2659)	102		11.4% (31/272)	4.8% (34/707)
Kelly et al.^3^	M alone	4.20%	9.18	Biopsy result and 1-year follow-up	39% (23 / 59)	N/A
	M plus US	9.60%	20.85		34.3% (46/134)	N/A
*Patients with dense breasts and negative mammography*						
Destounis et al.^39^	M (–) → US		20	Biopsy result	18% (18/100)	N/A
Klevos et al.^46^	M (–) → US		66	Biopsy result and 12-month follow-up	N/A	N/A
Kim et al.^45^	M (–) → US		Total: 46 Initial US: 96 Non-initial US: 23	Biopsy result	Total: 6.9% (9/131) Initial US: 4.5% (4/89) Non-initial US: 11.9% (5/42)	Total: 1.1% (9/831) Initial US: 0.9% (4/471) Non-initial US: 1.4% (5/360)
Weigert et al.^33^	M (–) → US		15.9	Biopsy result	Year 1: 7.3% (11/151)^a^ Year 2: 5.0% (9/180)^a^ Year 3: 7.4% (11/148)^a^ Year 4: 18.9% (10/53)^a^	Total: 2.9% (41/1400) Year 1: 3.4% (11/325) Year 2: 2.6% (9/348) Year 3: 3.5% (11/316) Year 4: 2.4% (10/411)
Chang et al.^37^	M (–) → US		84.8	Biopsy result and 12-month follow-up	5.95% (5/84)^a^	N/A
Girardi et al.^41^	M (–) → US		19	Biopsy result and at least 12-month follow-up	N/A	N/A
Hooley et al.^43^	M (–) → US		56.7 (BI-RADS category 3 + 4)	Biopsy result	5.6% (3/54 lesions) 6.5% (3/46 patients) (BI-RADS category 4)	N/A
Leong et al.^48^	M (–) → US		99	Biopsy result	14.3% (2/14)	N/A
Corsetti et al.^28^	M (–) → US			Biopsy result	N/A	7.5% (32/427)
Youk et al.^50^	M (–) → US		96.9	Biopsy or at least 2-year follow-up	33.9% (38/112)	N/A
Crystal et al.^38^	M (–) → US		25	Biopsy result	18.4% (7/38)	7.8% (7/90)
Kaplan^44^	M (–) → US		30	Biopsy result	11.8% (6/51)	N/A
Buchberger^35^	M (–) → US			Biopsy result	N/A	29.6% (37/125)

*M* mammogram, *US* ultrasound, *PPV3* positive predictive value of all biopsies performed, *PPV1* positive predictive value among cases that have positive results, *N/A* not available.

^a^PPV2, positive predictive value of all biopsy recommendations.

On the other hand, thirteen other studies included women with dense breasts and negative results on initial mammogram, and subsequently received additional US examination by handheld US (HHUS).^28,33,35,37–39,41,43–46,48,50^ In these studies, 196 out of 50,350 participants were diagnosed with malignancies confirmed by biopsy. Four of the studies that evaluated follow-up US were conducted in Far Eastern countries (Korea and Singapore) (Table 1).^37,45,48,50^ While Giuliano et al.^42^ adopted the Wolf classification of 50% or greater breast density for the definition of dense breasts, patients of all other studies were of BI-RADS categories 2–4, with 17 studies focusing on patients with BI-RADS categories 3 or 4. Six of the 13 studies that focused on follow-up US in patients with initial negative mammography reported the presence of breast cancer risk factors among their patients^38,39,43,46,48,50^ (Supplementary Table S1). Four studies focused on patients with definite negative mammography results prior to follow-up US,^35,37,38,48^ while patients with suspicious mammography results were included additionally by other studies (Supplementary Table S2).

The specificity and sensitivity of the different methods reported in the studies are summarised in Table 3. To achieve homogeneity in screening strategy among studies, the included studies were stratified for those comparing M alone versus M + US, and those with follow-up US during meta-analysis evaluations.

**Table 3:** Sensitivity and specificity of indicated screening strategy in the included studies.

First author	Comparison	Number of patients	Sensitivity	Specificity
*Patients with dense breasts*				
Wilczek et al.^49^	M alone	1668	63.6% (7/11)	99% (1641/1657)
	M plus US	1668	100% (11/11)	98.4% (1630/1657)
Brem et al.^34^	M alone	15,318	73.2% (82/112)	85.4% (12,987/15,206)
	M plus US		100% (112/112)	72% (10,954/15,206)
Giger et al.^40^	M alone	185	57.5% (30/52)	78.1% (104/133)
	M plus US	185	74.1% (38/52)	76.2% (102/133)
Giuliano^42^	M alone	4076	76% (19/25)	98.21% (3977/4051)
	M plus US	3418	97.67% (42/43)	99.7%(3365/3375)
Korpraphong et al.^47^	M alone	14,483	91.3% (105/115)	98.6% (14173/14,368)
	M plus US		100% (115/115)	96.8% (13914/14,368)
Chae et al.^36^	M alone	12,505	54.55% (6/11)	95.85% (11974/12,494)
	M plus US	8359	100% (24/24)	94.8% (7908/8335)
Berg et al.^30^	M alone	2659	55.6% (20/36)	89.1% (2337/2623)
	M plus US		94.4% (34/36)	74.3% (1950/2623)
Kelly et al.^3^^a^	M alone	6425	40% (23/57)	95.15%
	M plus US	6425	81% (46/57)	98.7%
*Patients with negative mammogram and dense breasts*				
Destounis et al.^39^	M (–) → US	4898	100% (18/18)	N/A
Klevos et al.^46^	M (–) → US	394	N/A	N/A
Kim et al.^45^	M (–) → US	Total: 3,171 Initial US: 998 Non-initial US: 2173	Total: 100% (9/9) Initial US: 100% (4/4) Non-initial US: 100% (5/5)	Total: 74% (2340/3162) Initial US: 53% (527/994) Non-initial US: 83.6% (1813/2168)
Weigert et al.^33^	M (–) → US	Year 1: 2706 Year 2: 3351 Year 3: 4128 Year 4: 3331	Total: 97.6% (41/42) Year 1: 91.7% (11/12) Year 2: 100% (9/9) Year 3: 100% (11/11) Year 4: 100% (10/10)	Total: 89.9% (12,115/13,474) Year 1: 88.3% (2380/2694) Year 2: 89.9% (3003/3342) Year 3: 92.6% (3812/4117) Year 4: 87.9% (2920/3321)
Chang et al.^37^	M (–) → US	990	100% (5/5)	91.9% (906/985)
Girardi et al.^41^	M (–) → US	9960	N/A	N/A
Hooley et al.^43^	M (–) → US	935	100% (3/3)	N/A
Leong et al.^48^	M (–) → US	141	100% (2/2)	88.5% (92/104)
Corsetti et al.^28^	M (–) → US	3356	86.7%^b^	N/A
Youk et al.^50^	M (–) → US	1507	88.4% (38/43)	N/A
Crystal et al.^38^	M (–) → US	1517	100% (7/7)	94.5% (1427/1510)
Kaplan et al.^44^	M (–) → US	1862	N/A	N/A
Buchberger^35^	M (–) → US	8103	92.5% (37/40)	75.9% (277/365)

*M* mammogram, *US* ultrasound, *N/A* not available.

^a^This study was not included for meta-analysis because the specificity was derived based on recalls.

^b^Screening sensitivity = cancers detected at screening/cancers detected at screening plus interval cancers.

### Meta-analysis

#### M alone versus M+US in patients with dense breasts

**Fig. 2:**
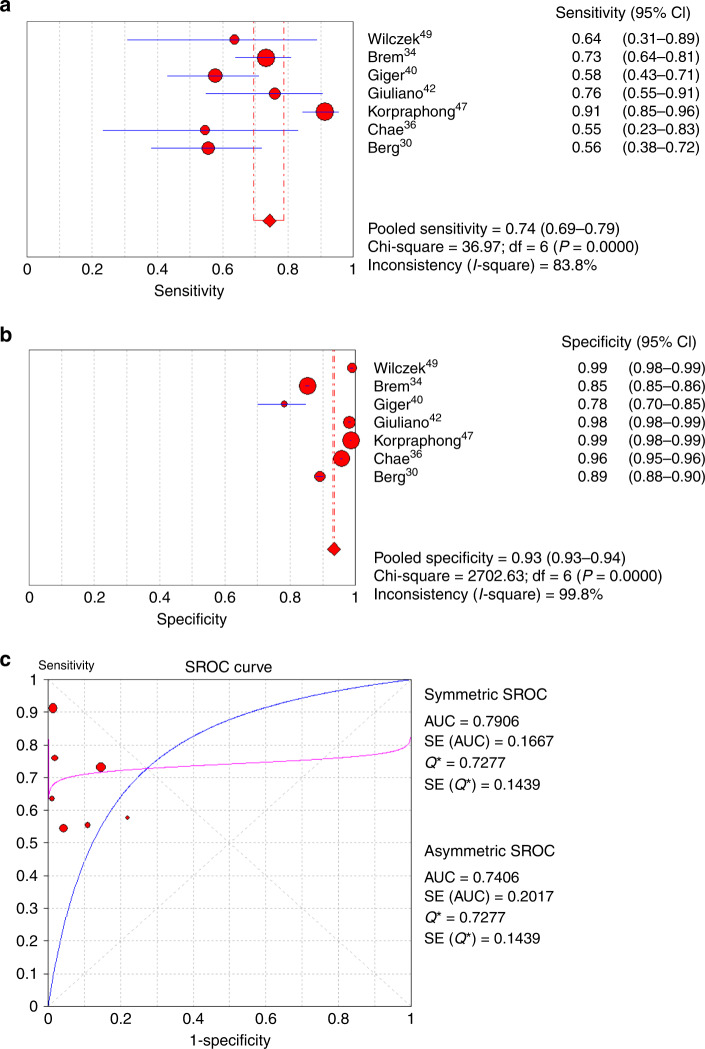
Meta-analysis of cancer diagnostic yield of mammography alone in patients with dense breast. **a** Sensitivity, **b** specificity and **c** summary of the ROC curve. ROC receiver-operating characteristics, SROC summary ROC, CI confidence interval, AUC area under SROC, SE standard error.

**Fig. 3:**
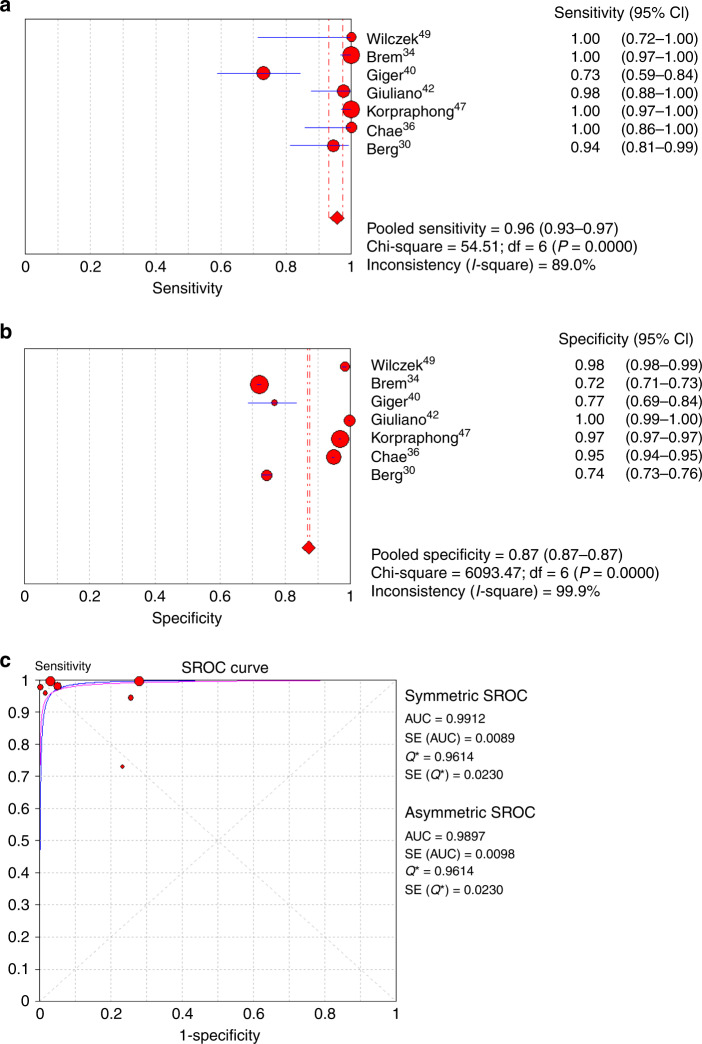
Meta-analysis of cancer diagnostic yield of mammography plus ultrasound in patients with dense breast. **a** Sensitivity, **b** specificity and **c** summary of the ROC curve. ROC receiver-operating characteristics, SROC summary ROC, CI confidence interval, AUC area under SROC, SE standard error.

Seven of the eight studies provided complete sensitivity and specificity data.^30,34,36,40,42,47,49^ The sensitivity of M alone for cancer detection ranged from 40% to 91.3%, and the specificity ranged from 78.1% to 99.0% (Table 3). High heterogeneity was found among studies reporting sensitivity or specificity of either methods (*I*^2^ ranged from 83.8% to 99.9%, all *P* < 0.001, Figs. 2 and 3). For this reason, a random-effect model was used for meta-analysis. For M + US, the sensitivity for cancer detection ranged from 74.1% to 100.0%, and the specificity ranged from 72% to 99.7%. For all studies combined, the pooled sensitivity and specificity of M alone for cancer detection was 74% (95% CI: 0.69–0.79) and 93% (95% CI: 0.93–0.94), respectively (Fig. 2). On the other hand, the pooled sensitivity and specificity for M + US was 96% (95% CI: 0.93–0.97) and 87% (95% CI: 0.87–0.87), respectively (Fig. 3).

**Fig. 4:**
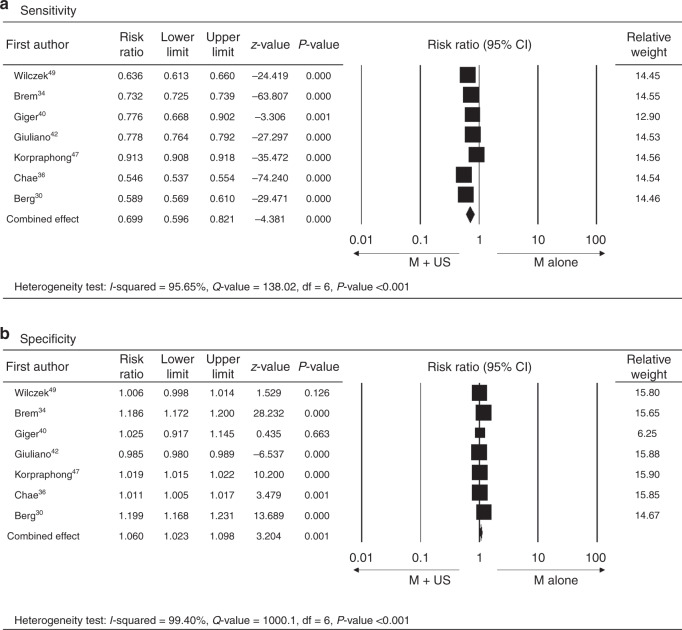
Meta-analysis of differences in cancer diagnostic yield between mammography alone and mammography plus ultrasound in patients with dense breast. **a** Sensitivity and **b** specificity. M alone, mammography alone, M + US mammography plus ultrasound, lower and upper limit, lower and upper bound of 95% confidence intervals (CI).

When comparing the diagnostic accuracy of cancer detection between M alone and M + US, the AUC value of the SROC curve from the combined effects among those studies showed that the M + US had better diagnostic efficacy of pooled sensitivity and specificity as compared with M alone (M + US vs. M alone, asymmetric SROC AUC value = 0.989 vs. 0.741) (Figs. 2c and 3c). In reflection to this finding, the meta-analysis of differences in the diagnostic yield of the two methods also showed that M + US might have higher sensitivity in cancer detection compared with mammography alone (M alone vs. M + US, RR = 0.699, 95% CI = 0.569–0.821, *P* < 0.001) (Fig. 4). The difference in specificity between M + US and M alone was shown significantly. However, the RR is represented close to 1 between two groups (RR = 1.060, 95% CI = 1.023–1.098, *P* = 0.001) (Fig. 4). The meta-analysis was performed by a random-effect model again, as high heterogeneity was found in the differences in diagnostic yield (difference in sensitivity: *I*^2^ = 95.65%, *P* < 0.001; difference in specificity: *I*^2^ = 99.4%, *P* < 0.001).

#### Follow-up ultrasound in patients with dense breasts and negative mammography

**Fig. 5:**
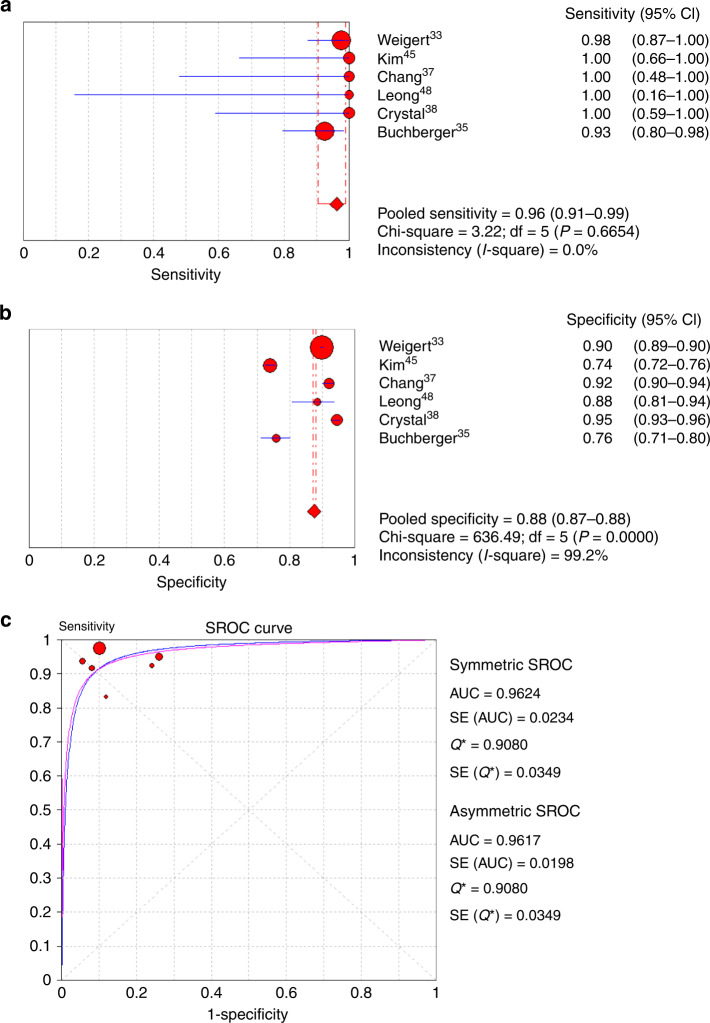
Meta-analysis of cancer diagnostic yield of follow-up ultrasound in patients with dense breast and negative mammography. **a** Sensitivity, **b** specificity and **c** summary of the ROC curve. ROC receiver-operating characteristics, SROC summary ROC, CI confidence interval, AUC area under SROC, SE standard error.

Six out of 13 studies with complete sensitivity and specificity data for the detection of malignancy by follow-up US in patients with negative mammography and dense breasts were included in the analysis.^33,35,37,38,45,48^ The sensitivity for cancer detection by follow-up US ranged from 88.4% to 100%, and specificity ranged from 74% to 94.5% (Table 3). A fixed-effect model was used for sensitivity, and a random-effect model used as high heterogeneity was found for specificity (sensitivity: *I*^2^ = 0%, *P* = 0.665; specificity: *I*^2^ = 99.2%, *P* < 0.001) (Fig. 5). Upon meta-analysis, the pooled sensitivity of cancer detection was found to be 96% (95% CI: 0.91–0.99) (Fig. 5a), and the pooled specificity was 88% (95% CI: 0.87–0.88) (Fig. 5b). The diagnostic accuracy (AUC) was derived as 0.962 (SE = 0.02) by asymmetric SROC (Fig. 5c).

### Subgroup analyses

To address potential confounding imposed by disease prevalence, US method and timing of follow-up US, subgroup analyses were conducted and summarised in Table 4.

**Table 4:** Subgroup analysis of studies given countries or US methods, respectively.

		Statistics				
	Number of studies	Sensitivity	Specificity	Asymmetric AUC (SE) of SROC	Risk ratio (95% CI) of sensitivity	Risk ratio (95% CI) of specificity
*Western countries*						
M alone	5	0.67 (0.61–0.73)	0.89 (0.88–0.89)	0.643 (0.118)	0.693 (0.636–0.755)*	1.079 (1.001–1.164)*
M + US	5	0.93 (0.90–0.96)	0.78 (0.78–0.79)	0.982 (0.023)	Reference	Reference
M (–) → US	3	0.95 (0.98–0.99)	0.90 (0.89–0.90)	0.970 (0.041)	–	
*Far Eastern countries*						
M alone	2	0.88 (0.81–0.93)	0.97 (0.97–0.98)	N/A	0.706 (0.426–1.169)	1.015 (1.008–1.023)*
M + US	2	1.00 (0.97–1.00)	0.96 (0.95–0.96)	N/A	Reference	Reference
M (–) → US	3	1.00 (0.79–1.00)	0.79 (0.77–0.80)	0.950 (0.035)	–	
*ABUS*						
M alone	4	0.69 (0.62–0.75)	0.89 (0.88–0.89)	0.671 (0.120)	0.722 (0.673–0.774)*	1.05 (0.969–1.137)
M + US	4	0.93 (0.89–0.96)	0.79 (0.78–0.79)	0.987 (0.021)	Reference	Reference
M (–) → US	0	–				
*HHUS*						
M alone	3	0.81 (0.74–0.87)	0.97 (0.96–0.97)	0.961 (0)	0.665 (0.446–0.99)*	1.067 (1.029–1.106)*
M + US	3	0.99 (0.96–1.00)	0.94 (0.94–0.94)	0.972 (0.333)	Reference	Reference
M (–) → US	6	0.96 (0.91–0.99)	0.88 (0.87–0.88)	0.962 (0.02)	–	
*First-round US screening*						
M alone	2	0.72 (0.64–0.80)	0.87 (0.86–0.87)	N/A	0.683 (0.595–0.784)*	1.092 (0.930–1.284)
M + US	2	1.00 (0.97–1.00)	0.75 (0.74–0.75)	N/A	Rreference	Reference
M (–) → US	3	1.00 (0.71–1.00)	0.73 (0.71–0.75)	0.944 (0.039)	–	

*M* mammogram, *US* ultrasound, *ABUS* automated breast US, *HHUS* handheld US, *N/A* not available, *ROC* receiver-operating characteristic, *SROC* summary ROC, *CI* confidence interval, *AUC* area under SROC, *SE* standard error.

*P value <0.05.

In studies conducted in either Western^3,10,30,34,40,42,49^ or Far Eastern^36,47^ countries, the diagnostic accuracy suggested that M + US had higher sensitivity but lower specificity compared with M alone (sensitivity: 0.93 vs. 0.67 in Western countries and 1.00 vs. 0.88 in the Far East; specificity: 0.78 vs. 0.89 in Western countries and 0.96 vs. 0.97 in Far East). The RR also showed that the M + US method had significantly higher sensitivity rate than M alone only in studies conducted in Western countries (RR = 0.69, 95% CI = 0.64–0.76, *P* < 0.001), while M + US method had a lower specificity rate than M alone in both Western and in Far Eastern countries (RR = 1.08, 95% CI = 1.00–1.16, *P* = 0.048 in Western countries; RR = 1.015, 95% CI = 1.008–1.023, *P* < 0.001 in the Far East). With regard to the US method, the diagnostic outcomes suggest that among studies adopting either ABUS^34,40,42,49^ or HHUS,^30,36,47^ M + US had higher sensitivity and lower specificity than M alone (sensitivity for ABUS: 0.93 vs. 0.69 for M + US vs. M alone; sensitivity for HHUS: 0.99 vs. 0.81 for M + US vs. M alone; specificity for ABUS: 0.79 vs. 0.89 for M + US vs. M alone; specificity for HHUS: 0.94 vs. 0.97 for M + US vs. M alone). The RR showed that the M + US method had significantly higher sensitivity rate than M alone, given that either ABUS or HHUS method was adopted (ABUS method: RR = 0.72, 95% CI = 0.67–0.77, *P* < 0.001; HHUS method: RR = 0.67, 95% CI = 0.45–0.99, *P* = 0.045), and significantly lower specificity rate than M alone only when HHUS method was performed (RR = 1.07, 95% CI = 1.03–1.11, *P* < 0.001) (Table 4). In studies that had data available specifically during the first-round US screening^34,49^ (Supplementary Table S2), M + US again had higher sensitivity and lower specificity than M alone (sensitivity: 1.00 vs. 0.72, RR = 0.683, *P* < 0.05; specificity: 0.75 vs. 0.87, RR = 1.09, *P* value insignificant).

For the six studies adopting follow-up US in patients with negative mammography and dense breasts, the sensitivity and specificity of the screening strategy were 0.95 and 0.90, respectively, for studies conducted in Western countries,^33,35,38^ and 1.00 and 0.79, respectively, for Far Eastern countries.^37,45,48^ The asymmetric AUC of SROC for Western countries was 0.97 (SE = 0.041); an asymmetric AUC of SROC = 0.950 (SE = 0.035) was found for studies conducted in Far Eastern countries. Regarding the US method, all six eligible studies were conducted using HHUS method,^33,35,37,38,45,48^ and the data were in line with the main results. Three studies evaluating follow-up US presented specific results for first-round screening^37,45,48^ (Supplementary Table S2), and the pooled sensitivity and specificity were 1.00 and 0.73, respectively, with an asymmetric AUC of SROC of 0.94 (Table 4).

Overall, the results of the subgroup analyses and the main meta-analysis exhibited similar trends.

### Sensitivity analysis among studies

Sensitivity analyses were performed using the leave-one-out approach in which the meta-analyses of cancer detection outcomes were performed with each study removed in turn. The results are summarised in Supplementary Tables S3 and S4. The direction and magnitude of combined estimates did not vary markedly with the removal of most of the studies, indicating that each of the meta-analyses had good reliability, and the data were not overly influenced by each study.

### Quality assessment

**Fig. 6:**
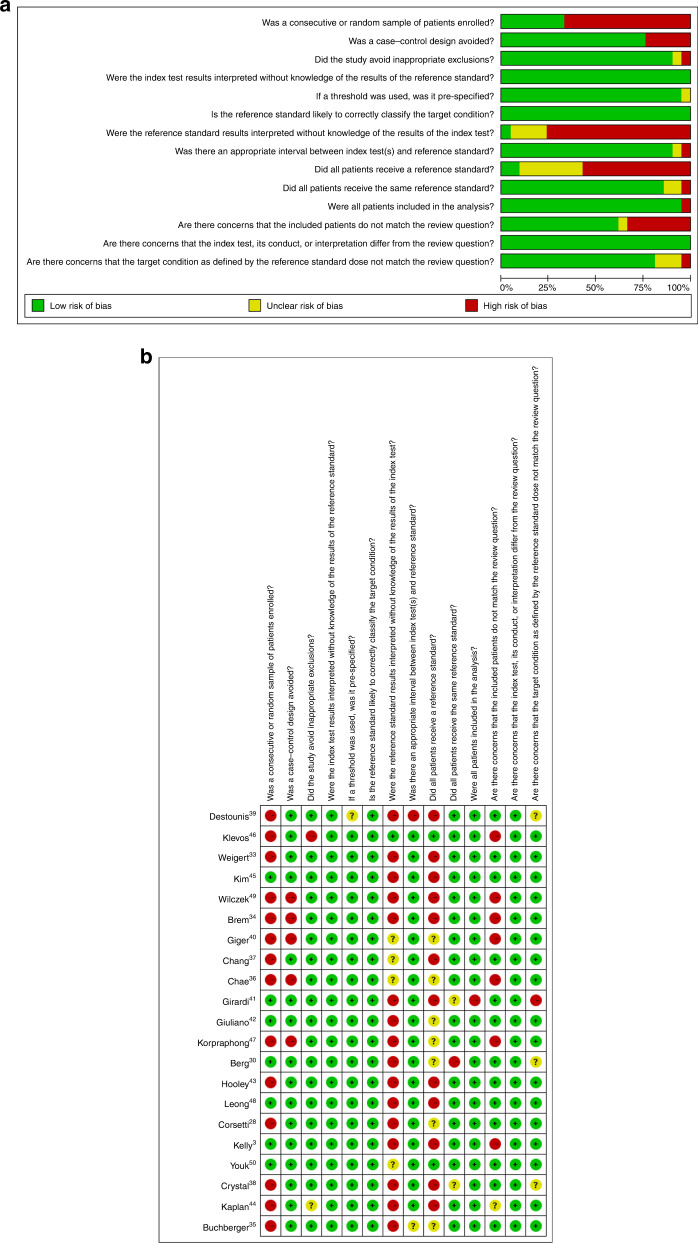
Quality assessment. **a** The summary of bias of the 21 studies; **b** risk of bias for each included study.

The quality assessments of included diagnostic accuracy studies are shown in Fig. 6. The quality assessment of the included studies indicated that the quality of the studies was acceptable, except for the retrospective design and the reference standard used by studies; the risk of bias mainly resulted from lack of enrolled-patient randomisation, index test masking and available reference standard in a number of studies.

## Discussion

This systematic review and meta-analysis examined and compared the diagnostic yield and accuracy of US as an adjunct to mammography with mammography alone for the screening of breast cancer in women with dense breasts. For general participants with dense breasts, the combined sensitivity of M + US for breast cancer was significantly higher than that of M alone (96% and 74%, respectively; RR = 0.699, *P* < 0.001). The combined specificity of M + US for breast cancer in the general female population with dense breasts was slightly lower than that of M alone (87% vs. 93%, respectively; RR = 1.06, *P* = 0.001). In contrast, in women with dense breasts and initially negative in mammography, the follow-up ultrasonography had high sensitivity (96%) and specificity (88%). Subgroup analyses with data stratified by study country, US method and first-round US further supported the main findings, suggesting that adjunctive US is beneficial for detecting breast cancer in women with dense breasts, albeit with an expected but tolerable sacrifice in detection specificity.

One meta-analysis published by Rebolj et al.^51^ examined the rate of breast cancer detected only by US versus that detected by multimodal screening methods (mammography with or without US). The authors found that the proportion of cancers detected only by US was 0.29 (95% CI: 0.27–0.31) of all detected cancers, and this translated to approximately 40% increased breast cancer detection compared with other screening methods. Furthermore, follow-up US additionally contributed to 3.8 (95% CI: 3.4–4.2) screen-detected cases per 1000 mammography-negative women. Despite these findings, US was not recommended by the authors to be a stand-alone screening method, but rather as a supplemental tool. It was difficult to correlate the findings reported by Rebolj et al.^51^ to our study, as neither the comparisons nor the outcomes of interests (M alone vs. M + US, and diagnostic yield in our case) were comparable between the two studies. Moreover, a fixed-effect model was adopted by Rebolj et al.^51^ disregarding the varied screening strategy and target population among their included studies, while a random-effect model was preferred in the current meta-analysis accompanied by study stratification.

Previously published systematic reviews have examined the usefulness of adding US to mammography screening for women with dense breasts. A 2009 review by Nothacker et al.^52^ only identified 6 cohort studies of intermediate-level evidence (3b) (no RCTs or other systematic reviews were identified). A more recent systematic review by Scheel et al.^53^ identified 12 studies, and concluded that there was consistent evidence that adjunctive US screening detects more invasive cancers compared with mammography alone in women with dense breasts, but there was no evidence to support that adjunctive US screening was associated with reduced long-term breast cancer mortality.^53^ In contrast to our study, the diagnostic outcomes of M + US did not receive individual review from follow-up US by Scheel et al.^53^. Furthermore, Scheel et al.^53^ study did not evaluate diagnostic yield by meta-analyses, which was also likely due to the disparate screening methodology adopted by studies included in the systematic review.^53^ A 2016 systematic review of supplemental screening for breast cancer in women with dense breasts done for the United States Preventive Services Task Force concluded that supplemental US screening increases the cancer detection rate, but was associated with an increase in the false-positive rate, and the impact on long-term breast cancer outcomes was unclear.^54^

The detection and differentiation of malignant microcalcifications in dense breast tissue are a particular issue of concern, but traditional radiologist-based interpretation of US imaging remains limited in providing an immediate solution.^21,22^ Computer-aided automatic reporting systems have been enthusiastically evaluated,^55,56^ and their implementation in future mammography screening may achieve greater diagnostic accuracy of microlesions in dense breasts. Furthermore, the adjunctive use of tomosynthesis in mammography-negative patients has been tested prospectively, and shown to have exhibited less false-positive results in contrast to supplemental US.^57^ In reflection, supplemental US screening for women with dense breasts was found to produce relatively small survival benefits, despite substantial increase in costs in a review using data from large medical databases and extensive literature search.^58^ Although outside the context of the current meta-analysis, the cost-effectiveness of US performed in the present fashion as a supplemental or follow-up screening for breast cancer should be carefully considered.

In the current meta-analysis, the subjective disparity and observer variability of US in each study could not be clearly distinguished, and thus may confound the findings. The image acquisition and interpretation of US are highly operator-dependent, and for this reason, computer-aided diagnosis systems have been rigorously developed in order to facilitate efficient interpretation, and improve the diagnostic accuracy in identifying malignant breast lesions.^59^ In addition, the observed differences among studies may depend on differences in learning curves, individual radiologic experience and the way protocols and reports are filled out. The low PPV reported by Brem et al.^34^ could be a result of the ABUS readout protocol, where the radiologist interpretation time was 2.9 min and evidently lower than that of Wilczek et al.^49^ In particular, low breast cancer rates in the Asian population may explain why Chae et al. reported low PPV values.^36^

Apart from the bias presented in the risk evaluations, the findings in the current meta-analyses may also be subjected to influence from heterogeneity among study design, patient characteristics, follow-up period and other details in the respective studies. Giger et al.^40^ performed an enriched-reader study involving 17 radiologists from different types of health facilities; thus, the readout performance or enrolled population may not be comparable to the real-world scenario.^10,60^ Leong et al.^48^ involved only one medical centre in their study, reporting a sensitivity of 100% since no false-negative cases were found after 1 year of follow-up of participants with BI-RADS assessment category 1 or 2 under mammogram and categories U1–U4 under US assessment.^48^ In Corsetti’s study in 2011,^28^ all subjects with negative screening mammograms and with dense breasts had bilateral breast US, and reported a screening sensitivity (86.7%) calculated by dividing cancers detected at screening with cancers detected at screening plus interval cancers occurring over 365 days for this study. Therefore, the variation of sensitivity of additional US ranging from 86.7% to 100% in the subgroup of patients with dense breasts and negative in mammography might result from heterogeneity in sample size and definition of true-positive cases.

There are limitations of this analysis that need to be considered in the interpretation of the results. The study design of most included studies in the analyses was retrospective rather than randomised head-to-head comparisons. Although the quality of the studies was found to be adequate, and the sensitivity analysis indicated that the results were robust, heterogeneity was detected among the studies. A number of studies evaluating the diagnostic effectiveness of follow-up US included patients who had initial suspicious rather than negative mammography results (Supplementary Table S2), and thus the effect imposed by prevalent cases could not be completely ruled out. In addition, we relied on breast-density results reported by the individual studies, and did not examine or stratify patients based on the actual breast density in the participants of the individual studies. Moreover, we did not take into account the mammography and US technical or instrumental differences among individual studies.

## Conclusions

The results of this systematic review and meta-analysis suggest that the addition of US to mammography screening of women with dense breasts improves the sensitivity for the detection of breast cancer, despite a slightly decreased specificity. Follow-up US also had good diagnostic sensitivity and specificity for screening women with dense breasts and negative mammogram findings. Future prospective studies designed to evaluate US as an adjunct or follow-up screening method to mammography in women with dense breasts are needed to confirm the results from our meta-analysis. Enrolment of specific high-risk populations should be further considered to identify those that may benefit from adjunctive US screening for breast cancers most cost-effectively, and reduce the number of recall or false-negative biopsies performed.

## Supplementary information

Supplementary Tables

## Data Availability

All data generated within this study are available from the corresponding author on request.
