# Supplementary material for: Supplemental breast cancer-screening ultrasonography in women with dense breasts: a systematic review and meta-analysis
Source: Br J Cancer. 2020 Jun 12;123(4):673–88. doi: 10.1038/s41416-020-0928-1 (PMC7434777; doi:10.1038/s41416-020-0928-1)
Supplement: Supplementary file 1 — Supplementary Tables [file 41416_2020_928_MOESM1_ESM.docx]

**Supplementary Tables**

**Table S1 Summary of the risk factors considered in each of the study.**

| **First author**  **(publication year)** | >=1  risk factors | Mutation in BRCA1 or BRCA2 | | History of Hormone therapy | History of biopsy | Personal history of breast cancer | Family history of breast cancer | Intermediate risk^@^ | High or very strong  risk# |
| --- | --- | --- | --- | --- | --- | --- | --- | --- | --- |
| ***Patients with dense breasts*** |  |  | |  |  |  |  |  |  |
| Wilczek (2016) |  |  | | 14.9% | 3.8% | 0.2% | 3.5% |  |  |
| Brem (2015) |  | 1.0% | | 31.2% |  | 3.6% | 44.8% |  |  |
| Giger (2016) |  |  | |  |  |  |  |  |  |
| Giuliano (2013) |  | 0% | |  |  | 0% | 0% |  |  |
| Korpraphong (2014) |  |  | |  |  |  |  |  |  |
| Chae (2013) |  |  | |  |  |  |  |  |  |
| Berg (2012) |  | 0.9% | |  |  | 53.1% |  |  |  |
| Kelly (2010)* |  | 0.10% | | 41% |  | 10% | 59% |  |  |
| ***Patients with negative mammogram and dense breasts*** | | | | | | | |  |  |
| Destounis (2017)* | 67% | |  |  |  |  | 54.4% |  |  |
| Klevos (2017)* |  | | 0% |  |  |  | 0% |  |  |
| Kim (2016) |  | |  |  |  |  |  |  |  |
| Weigert (2017) |  | |  |  |  |  |  |  |  |
| Chang (2014) |  | |  |  |  |  |  |  |  |
| Girardi (2013)* |  | |  |  |  |  |  |  |  |
| Hooley (2012)* |  | |  |  |  |  |  | 15.9% | 9.3% |
| Leong (2012) |  | |  |  |  | 5% | 20.5% |  |  |
| Corsetti (2011)* |  | |  |  |  |  |  |  |  |
| Youk (2011)* |  | |  |  |  | 61.8% |  |  |  |
| Crystal (2003) |  | |  |  |  | 20.9% |  |  |  |
| Kaplan (2001)* |  | |  |  |  |  |  |  |  |
| Buchberger (2000) |  | |  |  |  |  |  |  |  |

Blank entries indicate value not available.

*, study not included for meta-analysis.

# High or strong risk: premenopausal mother or sister, or multiple premenopausal first-degree relatives with breast cancer, BRCA positive

@ Intermediate risk: postmenopausal mother or sister with breast cancer or personal history of breast cancer

**Table S2 Additional details of screening method and patient population in studies included for meta-analysis.**

| **First author (publication year)** | **Was it the first round of US or not?** | **Were mammography and US performed in the same population or not?** | **When US was performed after a negative mammogram, was the mammogram really negative or suspicious?** |
| --- | --- | --- | --- |
| ***Patients with dense breasts*** | | | |
| Wilczek (2016) | Yes | Yes | - |
| Brem (2015) | Yes | Yes | - |
| Giger (2016) | NR | Yes | - |
| Giuliano (2013) | NR | Overlap (all had mammography, only 3418 had US additionally; specific data not available) | - |
| Korpraphong (2014) | NR | Yes | - |
| Chae (2013) | NR | Overlap (all had mammography, only 8359 had US additionally; specific data not available) | - |
| Berg (2012) | Not all; specific data not available | Yes | - |
| ***Patients with negative mammogram and dense breasts*** | | | |
| Kim (2016) | Not all; first-round data available | Yes | Could be negative or suspicious (BI-RADS score not specified; specific data not available) |
| Weigert (2017) | Not all; specific data not available | Yes | Could be negative or suspicious (specific data not available) |
| Chang (2014) | Yes | Yes | Negative (BI-RADS score 1 or 2; suspicious were excluded) |
| Leong (2012) | Yes | Yes | Negative (BI-RADS score 1 or 2) |
| Crystal (2003) | NR | Yes | Negative (suspicious were excluded) |
| Buchberger (2000) | NR | Yes | Negative (suspicious were excluded) |

NR, not reported.

**Table S3 Sensitivity analysis among studies that presented diagnostic yield of mammography alone or plus ultrasound in patients with dense breasts screened and that of follow-up ultrasound in patients with negative mammogram.**

|  | **Statistics with study removed** | | | |
| --- | --- | --- | --- | --- |
| **Removed studies** | **Sensitivity** | **Specificity** | **AUC (SE)** | **Asymmetric AUC (SE)** |
| **First author (Publication year)** |  |  |  |  |
| ***M alone in patients with dense breasts*** |  |  |  |  |
| Wilczek (2016) | 0.75 (0.70 - 0.79) | 0.93 (0.93 - 0.93) | 0.7644 (0.2341) | 0.7048 (0.2746) |
| Brem (2015) | 0.75 (0.69 - 0.80) | 0.97 (0.97 - 0.97) | 0.7753 (0.2507) | 0.7231 (0.2879) |
| Giger (2016) | 0.77 (0.72 - 0.82) | 0.93 (0.93 - 0.94) | 0.8731 (0.1429) | 0.8451 (0.1999) |
| Giuliano (2013) | 0.74 (0.69 - 0.79) | 0.93 (0.93 - 0.93) | 0.7946 (0.1799) | 0.7515 (0.2216) |
| Korpraphong (2014) | 0.66 (0.60 - 0.72) | 0.91 (0.91 - 0.92) | 0.6999 (0.1159) | 0.6559 (0.1155) |
| Chae (2013) | 0.75 (0.70 - 0.79) | 0.93 (0.92 - 0.93) | 0.7789 (0.1814) | 0.7169 (0.2105) |
| Berg (2012) | 0.76 (0.71 - 0.81) | 0.94 (0.93 - 0.94) | 0.8268 (0.1577) | 0.7790 (0.2055) |
| Kelly (2010) | Not included for meta-analysis | | | |
| ***M+US in patients with dense breasts*** |  |  |  |  |
| Wilczek (2016) | 0.96 (0.93 - 0.97) | 0.87 (0.86 - 0.87) | 0.9904 (0.0120) | 0.9894 (0.0113) |
| Brem (2015) | 0.94 (0.90 - 0.96) | 0.95 (0.94 - 0.95) | 0.9909 (0.0100) | 0.9840 (0.0270) |
| Giger (2016) | 0.99 (0.97 - 1.00) | 0.87 (0.87 - 0.87) | 0.9962 (0.0031) | 0.9920 (0.0072) |
| Giuliano (2013) | 0.95 (0.93 - 0.97) | 0.86 (0.86 - 0.86) | 0.9767 (0.0256) | 0.9710 (0.0451) |
| Korpraphong (2014) | 0.94 (0.90 - 0.96) | 0.83 (0.82 - 0.83) | 0.9883 (0.0120) | 0.9837 (0.0174) |
| Chae (2013) | 0.95 (0.93 - 0.97) | 0.85 (0.85 - 0.86) | 0.9908 (0.0108) | 0.9893 (0.0118) |
| Berg (2012) | 0.96 (0.93 - 0.98) | 0.88 (0.88 - 0.88) | 0.9934 (0.0074) | 0.9930 (0.0074) |
| Kelly (2010) | Not included for meta-analysis | | | |
| ***Follow-up US in patients with dense breasts and negative mammogram*** |  |  |  |  |
| Destounis (2017) | Not included for meta-analysis | | | |
| Klevos (2017) | Not included for meta-analysis | | | |
| Weigert (2017) | 0.95 (0.87 - 0.99) | 0.82 (0.81 - 0.83) | 0.9601 (0.0226) | 0.9439 (0.0388) |
| Kim (2016) | 0.96 (0.90 - 0.99) | 0.90 (0.90 - 0.91) | 0.9622 (0.0250) | 0.9621 (0.0270) |
| Chang (2014) | 0.96 (0.90 - 0.99) | 0.87 (0.87 - 0.88) | 0.9603 (0.0305) | 0.9602 (0.0286) |
| Girardi (2013) | Not included for meta-analysis | | | |
| Hooley (2012) | Not included for meta-analysis | | | |
| Leong (2012) | 0.96 (0.90 - 0.99) | 0.87 (0.87 - 0.88) | 0.9742 (0.0216) | 0.9645 (0.0285) |
| Corsetti (2011) | Not included for meta-analysis | | | |
| Youk (2011) | Not included for meta-analysis | | | |
| Crystal (2003) | 0.96 (0.90 - 0.99) | 0.87 (0.86 - 0.87) | 0.9495 (0.0378) | 0.9475 (0.0518) |
| Kaplan (2001) | Not included for meta-analysis | | | |
| Buchberger (2000) | 0.99 (0.92 - 1.00) | 0.88 (0.87 - 0.88) | 0.9684 (0.0188) | 0.9647 (0.0326) |

Abbreviations: AUC, area under the summary receiver operating characteristic (SROC) curve; M alone, mammography alone; M+US, Mammography plus ultrasound; SE, standard error.

**Table S4 Sensitivity analysis among studies presenting differences in cancer diagnostic yield between mammography alone and mammography plus ultrasound in patients with dense breasts.**

|  | **Statistics with study removed** | | | | |
| --- | --- | --- | --- | --- | --- |
| **Removed study First author (publication year)** | **Risk ratio** | **Lower limit** | **Upper limit** | **z-value** | **p-value** |
| **Sensitivity** |  |  |  |  |  |
| Wilczek (2016) | 0.710 | 0.598 | 0.845 | -3.876 | <.001 |
| Brem (2015) | 0.694 | 0.555 | 0.868 | -3.200 | 0.001 |
| Giger (2016) | 0.688 | 0.580 | 0.817 | -4.260 | <.001 |
| Giuliano (2013) | 0.687 | 0.570 | 0.828 | -3.947 | <.001 |
| Korpraphong (2014) | 0.667 | 0.584 | 0.762 | -5.961 | <.001 |
| Chae (2013) | 0.729 | 0.639 | 0.832 | -4.704 | <.001 |
| Berg (2012) | 0.720 | 0.607 | 0.853 | -3.783 | <.001 |
| **Specificity** |  |  |  |  |  |
| Wilczek (2016) | 1.070 | 1.026 | 1.116 | 3.164 | 0.002 |
| Brem (2015) | 1.035 | 1.011 | 1.059 | 2.926 | 0.003 |
| Giger (2016) | 1.062 | 1.024 | 1.102 | 3.218 | 0.001 |
| Giuliano (2013) | 1.074 | 1.028 | 1.123 | 3.210 | 0.001 |
| Korpraphong (2014) | 1.067 | 1.013 | 1.125 | 2.451 | 0.014 |
| Chae (2013) | 1.069 | 1.023 | 1.118 | 2.964 | 0.003 |
| Berg (2012) | 1.038 | 1.001 | 1.075 | 2.026 | 0.043 |
